# Comparative Metabolome and Transcriptome Analyses Reveal the Regulatory Mechanism of Purple Leafstalk Production in Taro (*Colocasia esculenta* L. Schott)

**DOI:** 10.3390/genes15010138

**Published:** 2024-01-22

**Authors:** Shizheng Jiang, Juxian Guo, Imran Khan, Mohammad Shah Jahan, Kang Tang, Guihua Li, Xian Yang, Mei Fu

**Affiliations:** 1 Guangdong Key Laboratory for New Technology Research of Vegetables, Vegetable Research Institute, Guangdong Academy of Agricultural Sciences, Guangzhou 510642, China; jiangshizheng2021@163.com (S.J.); guojuxian@gdaas.cn (J.G.); dh18006@yzu.edu.cn (I.K.); 20203137146@stu.scau.edu.cn (K.T.); liguihua@gdaas.cn (G.L.); 2 College of Horticulture, South China Agricultural University, Guangzhou 510642, China; yangxian@scau.edu.cn; 3 Department of Horticulture, Faculty of Agriculture, Sher-e-Bangla Agricultural University, Dhaka 1207, Bangladesh; shahjahansau@gmail.com

**Keywords:** taro leafstalk, metabolome, transcriptome, anthocyanins

## Abstract

Taro is a plant in the *Araceae* family, and its leafstalk possesses significant botanical and culinary value owing to its noteworthy medicinal and nutritional attributes. Leafstalk colour is an essential attribute that significantly influences its desirability and appeal to both breeders and consumers. However, limited information is available about the underlying mechanism responsible for the taro plant’s colouration. Thus, the purpose of the current study was to elucidate the information on purple leafstalks in taro through comprehensive metabolome and transcriptome analysis. In total, 187 flavonoids, including 10 anthocyanins, were identified. Among the various compounds analysed, it was observed that the concentrations of five anthocyanins (keracyanin chloride (cyanidin 3-O-rutinoside chloride), cyanidin 3-O-glucoside, tulipanin (delphinidin 3-rutinoside chloride), idaein chloride (cyanidin 3-O-galactoside), and cyanidin chloride) were found to be higher in purple taro leafstalk compared to green taro leafstalk. Furthermore, a total of 3330 differentially expressed genes (DEGs) were identified by transcriptome analysis. Subsequently, the correlation network analysis was performed to investigate the relationship between the expression levels of these differentially expressed genes and the content of anthocyanin. There were 18 DEGs encoding nine enzymes detected as the fundamental structural genes contributing to anthocyanin biosynthesis, along with seven transcription factors (3 MYB and 4 bHLH) that may be promising candidate modulators of the anthocyanin biosynthesis process in purple taro leafstalk. The findings of the current investigation not only provide a comprehensive transcriptional code, but also give information on anthocyanin metabolites as well as beneficial insights into the colour mechanism of purple taro leafstalk.

## 1. Introduction

Taro leafstalk have high medicinal and edible value. The taro leafstalk not only contains an abundance of proteins, vitamins, and carbohydrates, but also contains other phytonutrients with antioxidant potential as well [1]. A purple taro leafstalk is more appealing to consumers due to its colour. Plants exhibit a variety of colours owing to the presence of several pigments, including chlorophyll, carotenoids, and anthocyanins [2,3]. Anthocyanins play a key role in the process of leaf colouration; these pigments are classified as flavonoids, are water-soluble, and are widely distributed throughout many plant species. Currently, the number of identified anthocyanins in nature exceeds 600, of which petunidin, delphinidin, pelargonidin, malvidin, peonidin, and cyanidin have been found to be the most common anthocyanins in plants [4]. The anthocyanin pigments present in seeds, vegetables, flowers, and fruits bestow vibrant colours, including purple, red, pink, and blue, upon diverse tissues and organs [5,6,7]. In addition, anthocyanins are beneficial for mitigating the detrimental effects induced by low temperature, water deficit, salinity, ultraviolet light, and low-phosphate stressors, and play a crucial role in attracting animals and insects that facilitate the processes of pollination and seed dissemination [8,9,10]. There is a recent trend of scientists considering anthocyanins as a potentially valuable dietary constituent owing to their pharmacological and biological properties, which encompass anti-aging, antioxidant activity, and cancer prevention features [11,12].

In higher plants, anthocyanin biosynthesis pathways have been widely explored alongside flavonoid pathways [13,14]. In general, phenylalanine is typically broken down enzymatically by phenylalanine ammonia-lyase (PAL), which starts the process of anthocyanin synthesis. Following that, 4-coumaroyl CoA is produced from cinnamic acid by 4-coumaroyl CoA ligase and cinnamate 4-hydroxylase (C4H). An intermediate, naringenin, is synthesised by chalcone synthase (CHS) and chalcone isomerase (CHI) from 4-coumarolyl-CoA and 3-malonyl-CoA. Additionally, the production of distinct coloured anthocyanidins is facilitated by enzymatic reactions involving flavonoid 3-hydroxylase (F3H), flavonoid 3′-hydroxylase (F3′H), flavonoid 3′5′-hydroxylase (F3′5′H), dihydroflavonol 4-reductase (DFR), and anthocyanidin synthase (ANS). The conversion of naringenin into the aforementioned anthocyanins is dependent upon the presence of these enzymes. Finally, after glycosylating by UDP-glucosyltransferases (UGTs), anthocyanins are transferred to vacuoles by glutathione S-transferases (GSTs). Additionally, the transfer of anthocyanins to vacuoles encompasses the participation of various transporter proteins, including MATE proteins and ABC transporters [15,16].

The regulation of anthocyanin biosynthesis involves an intricate interaction among several transcription factors (TFs). As an illustration, the transcriptional activation complex MBW comprises MYB, bHLH, and WD40 components, collectively responsible for the regulation of gene expression related to anthocyanin biosynthesis. Notably, MYB plays a crucial role in this complex [17]. Two distinct MYB types (R2R3- and R3-MYB) participate in a competitive process to form the MBW complex, thus exerting differential effects on the anthocyanin biosynthesis process [18,19]. In addition, it is probable that an independent R2R3-MYB transcription factor is involved in regulating the production of anthocyanins [20]. But the bHLH transcription factor creates complexes with WD40 and MYB proteins to effectively control the expression of the critical genes linked to anthocyanin biosynthesis [21]. In addition, WD40 plays a regulatory role in anthocyanin biosynthesis through its interactions with MYB and bHLH proteins [22]. In recent studies, other transcription factors, BBX [23,24,25], ERF [26,27], WRKY [28,29], and NAC [30,31], have been characterised and recognised to influence anthocyanin biosynthesis in plants.

In recent times, there has been a notable increase in the implementation of multi-omics and high-throughput genome sequencing approaches, resulting in the advancement of research on plant functional characteristics and the uncovering of unique metabolic pathways, making it possible to determine the pivotal genes associated with phenylpropanoid biosynthesis [32]. Integrated transcriptome and metabolome investigations have made it possible to pinpoint genes responsible for leaf colour, fruit development, and pigment accumulation [33,34]. Currently, the precise function and coordination of fundamental genes connected with anthocyanin biosynthesis in taro leafstalk remain poorly understood.

The current study used metabolomics and transcriptome technologies to discover DEGs and clarify alterations in anthocyanin production that are responsible for the colour shift in taro leafstalk. We examined the RNA-seq abundance and HPLC profiles of both green and purple taro leafstalks in order to look into the relationship between structural genes that encode the enzymes that promote anthocyanin production and the onset of pigmentation in taro leafstalk. Our study’s results provide an understanding of the role of functional genes and metabolites in the control of taro leafstalk colour, which has important ramifications for the progress of colour enhancement in taro and other pigmented vegetables.

## 2. Materials and Methods

### 2.1. Plant Materials

We cultivated two types of taro at the Vegetable Research Institute, Guangdong Academy of Agricultural Sciences, in March 2022, with controlled greenhouse conditions of 16 h light and 8 h dark photoperiods without any natural light. There was no significant difference between the two varieties except the colour. The purple (P) taro germplasms, named “Dinganyugan” (“DAYG”) were collected from Dingan, Hainan Province, China, and the green ones, named “Danzhouyugan” (“DZYG”) were collected from Danzhou, Hainan Province, China. After 45 days of growth, the epidermal tissue was separated from the leafstalk of taro with a blade, and liquid nitrogen was applied immediately to freeze the leafstalk. Samples were collected in the morning. In order to facilitate further analysis, plant samples were stored at −80 °C. For each variety, three biological replicates were set up, with one biological replicate containing five unique plants. 

### 2.2. Separation and Detection of Total Flavonoids in Taro Leafstalk

The frozen samples were ground into a fine powder using a mixer mill. Total flavonoids were extracted by weighing 100 mg of powder accurately and squeezing it into 500 μL of 80% methanol solution via a well vortex. Centrifugation (15,000× *g* for 10 min at 4 °C) was used to remove sediment from the extract. After diluting the supernatant with mass-spectrometry-grade water to a methanol content of 53% [35], the diluted sample was centrifuged for 20 min at 15,000× *g* and 4 °C. The supernatant was used to analyse the metabolome. Analysis of the extraction solution was carried out using an LC-ESI-MS/MS system (HPLC, SCIEX, Exion LC; MS, SCIEX, QTRAP 6500+).

### 2.3. RNA Extraction and Illumina Sequencing

RNA was extracted from tissue samples by grinding in liquid nitrogen and using Trizol reagent kits (Invitrogen, Carlsbad, CA, USA). The quality of the RNA was analysed using an Agilent 2100 Bioanalyzer (Agilent Technologies, Palo Alto, CA, USA). Mature mRNA in eukaryotes had a poly (A) tail, while other RNAs did not; oligo dT magnetic beads were used to specifically bind to the poly (A) tail of mRNA to remove other RNAs. Then fragmentation reagent was used to bind specifically to the poly (A) tail of mRNA in order to remove other RNAs. Transcription of short fragments into cDNA was performed using the NEBNext Ultra RNA Library Prep Kit for Illumina (NEB #7530, New England Biolabs, Ipswich, MA, USA). After repairing double-stranded cDNA, Illumina sequencing adapters were added. Gene Denovo Biotechnology Co. (Guangzhou, China) sequenced the constructed cDNA library using Illumina Novaseq6000.

### 2.4. RNA Data Analysis and Annotation

In order to acquire high-quality, clean reads for assembly and analysis, fastp (version 0.18.0) software was implemented [36]. For the alignment of the clean reads to the ribosome database, we utilised bowtie2 (version 2.2.8) [37]. Subsequently, uncategorised reads were employed for further transcriptome analysis through the exclusion of mapped reads found in the ribosomes. The HISAT2 software was used to align high-quality reads to taro genome with HISAT2 2.4 [38]. To evaluate the degree of gene expression, the fragments of transcript per kilobase per million mapped (FPKM) reads values were calculated [39,40]. In order to annotate the transcripts, several databases were used, including gene ontology (GO) and NCBI non-redundant (Nr), Kyoto Encyclopedia of Genes and Genomes (KEGG) and Pfam, and Swiss-Prot protein databases. DESeq2 was used to identify the DEGs between purple and green leafstalk to facilitate further analysis. 

### 2.5. Transcription Factor Analysis

The synthesis of anthocyanin is dependent on transcription factors (TFs), so the TFs expressed in each sample were determined. To retrieve all the putative transcription factors, a search was performed using the keyword “transcription factor” against the transcription annotation file, and both the number and type of TFs were counted accordingly. Subsequently, to address the relationship between the TFs and anthocyanin content, we also computed the Pearson correlation coefficient (PCC). The transcription factors with >0.9 scores according to the PCC were considered for further examination.

### 2.6. Validation RNA-Seq by Quantitative Real Time PCR

The RNAprep Pure Plant Plus Kit (Biomarker, Beijing, China) was used for the isolation of total RNA. The first-strand cDNA was made by utilizing the HiScript III RT SuperMix for qPCR (+gDNA wipe) (Vazyme, Nanjing, China). The CFX96 PCR machine from Bio-Rad was employed for the quantitative real-time (qRT)-PCR analysis, and ChamQ Universal SYBR qPCR Master Mix from Vazyme was chosen as the fluorescent additive. A reaction system was created with a total volume of 10 μL, including 5 μL qPCR Master Mix; 1 μL cDNA template; a downstream primer and an upstream primer, both of 0.3 μL; and 3.4 μL ddH_2_O. The primers used in this study for qRT-PCR were designed utilizing primer premier 5 software, and their details are listed in Table S1. To validate the results obtained from RNA-seq, we randomly chose a total of 8 DEGs for qRT-PCR. The actin gene was employed as an internal reference. The results of the qRT-PCR were computed using the 2^−ΔΔCT^ method.

### 2.7. Data Analysis

Differentially abundant metabolites (DAMs) were identified based on fold change >2 or <0.5 and *p* < 0.05. The genes that met the standards of a false discovery rate (FDR) < 0.05 and |log2 fold change| > 1 were designed as differentially expressed genes (DEGs) in the transcriptome. Three biological replicates were conducted for each experiment, and the data are displayed as the mean ± SD. Statistical analyses were performed using *t*-tests with GraphPad Prism 8.0.lnk, and *p* < 0.05 was considered to indicate significant differences. 

## 3. Results

### 3.1. Functional Analysis of Metabolites

**Figure 1:**
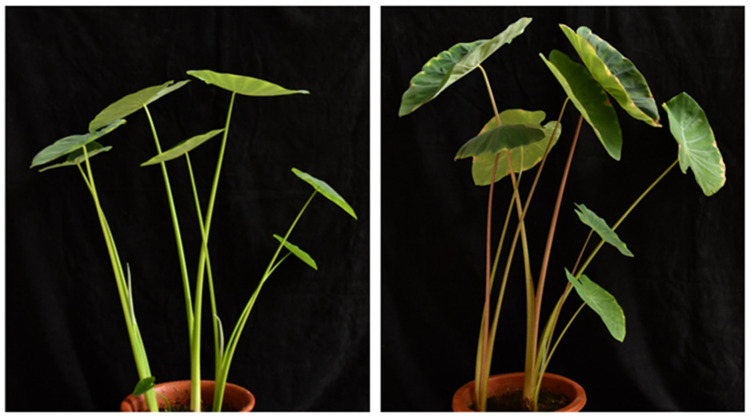
Phenotypes of 45-day-old purple and green varieties of taro.

**Figure 2:**
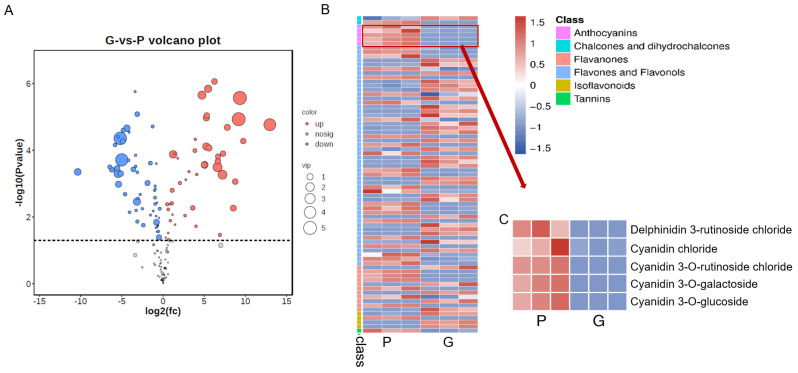
Volcano plot analysis and DAM analysis of metabolome. (**A**) Volcano of metabolite profile in purple and green taro leafstalk. (**B**) Heat map of DAM. (**C**) Heat map of differential DAA.

To investigate the anthocyanin components of taro leafstalk, we conducted metabolomics analysis using purple and green taro leafstalk (Figure 1). Both groups of metabolites showed clear separation between them with PC1 (61.5%) and PC2 (13.8%) (Figure S1A). In addition, it was observed that biological replicates of the same variety tended to cluster together, suggesting that the metabolomics data showed a high level of repeatability and reliability. There was an obvious difference in metabolic components for purple and green taro leafstalk, as shown by the data on the left side of Figure S1A and the right side of Figure S1A, respectively. Based on these metabolites, hierarchical heatmap clustering analysis (HCA) was carried out for all samples. Each variety was categorised separately, indicating that there were significant differences in metabolites between the two varieties (Figure S1B). In addition, the Pearson’s correlation coefficient also demonstrated an obvious difference between the two sample groups, which aligned with the findings of the principal component analysis (PCA) (Figure S1C). DAMs were identified according to the criteria mentioned earlier in purple taro leafstalk compared to green taro leafstalk, where 38 metabolites were found to be upregulated, whereas 37 metabolites were downregulated (Figure 2A, Table S2). A heatmap depicting the distribution of all DAMs (Figure 2B) revealed that these flavonoids were categorised into six distinct groups, namely, anthocyanins, chalcones, dihydrochalcones, flavanones, flavonoids, flavones, flavonols, isoflavones, and tannin. Among these DAMs, a total of five differentially abundant anthocyanins (DAAs) were identified, as shown in Figure 2C.

### 3.2. Analysis of Transcriptome Sequencing Quality

Through the utilisation of Illumina RNA-seq technology, a transcriptome study was conducted to investigate the comparative gene expression patterns of purple and green taro leafstalk. After eliminating reads comprising adaptors and N ratios exceeding 10%, a total of 41.2 G clean reads were acquired for both taro leafstalk varieties. As shown in Table S3, the GC content was between 50% and 52%, the Q20 percent (% of nucleotides with quality > 20) was greater than 96.46%, and the Q30 percent (% of nucleotides with quality values > 30) was greater than 91.03%. The results of the correlation coefficient analysis (Figure S2A) and the principal component analysis (PCA) (Figure S2B) demonstrated consistent expression patterns among the replicates. 

### 3.3. Analysis of DEGs in Purple Taro Leafstalk and Green Taro Leafstalk

**Figure 3:**
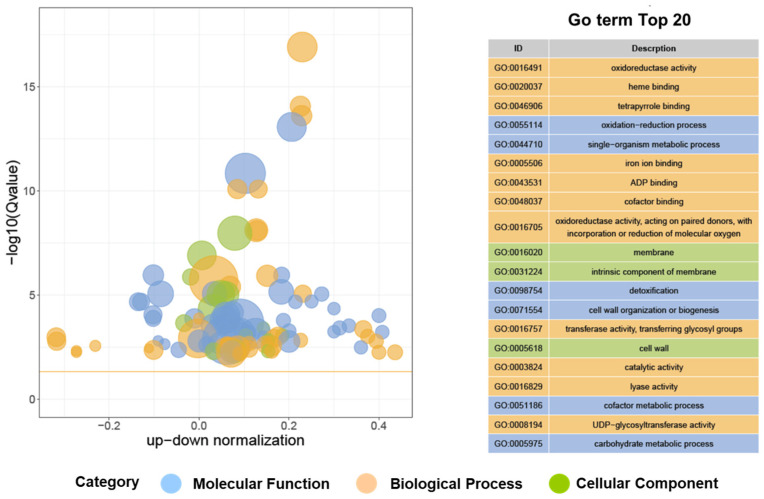
Top 20 GO enrichment analysis of all DEGs between purple and green taro leafstalks. The blue colour represents the molecular function. The orange colour represents the biological process, and the green represents the cellular component. The horizontal axis represents the proportion of the difference between the number of upregulated differentially expressed genes and the number of downregulated differentially expressed genes in the total differentially expressed genes.

**Figure 4:**
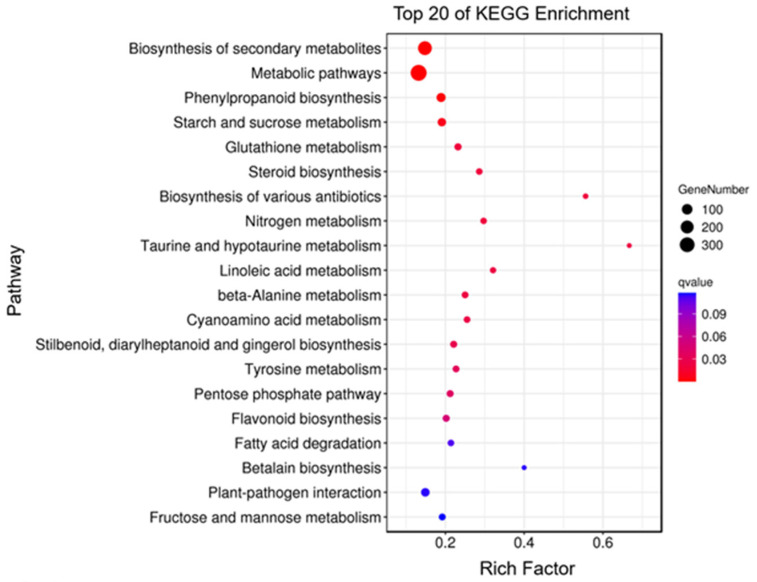
Enrichment of top 20 KEGG pathways of all DEGs between purple and green taro leafstalks.

Analysis of the DEGs discovered in the taro leafstalk revealed a total of 3330 DEGs in both the purple and green varieties. There were a total of 1761 genes which showed upregulation, whereas 1569 genes were downregulated (Table S4). Gene ontology (GO) mapping was utilised to provide a thorough annotation of all DEGs. We observed that DEGs were significantly enriched in 210 GO terms: 103 GO terms that referred to biological processes, 21 GO terms that referred to cellular components, and 86 GO terms that referred to molecular functions (Table S5). Figure 3 shows the top 20 GO categories of the most significant enrichment pathways. The predominant terms observed in the encompassed biological processes were “oxidation-reduction process” (GO:0055114), “detoxification” (GO:0098754), and “single-organism metabolic process” (GO:0044710). There was a significant enrichment in “membrane” (GO:0016020) and “intrinsic component of membrane” (GO:0031224) under the cellular component category. Based on the molecular functional classification, as depicted in Figure 3 and Table S5, a significant number of DEGs were linked to “heme binding” (GO:0020037) or “oxidoreductase activity” (GO:0016491). KEGG pathways were used to identify genes implicated in metabolic pathways in a subsequent investigation (Table S6). DEGs were highly enriched in a number of pathways, including “Biosynthesis of secondary metabolites” (Ko01110), Phenylpropanoid biosynthesis” (Ko00940) “Metabolic pathways” (Ko01100), “Glutathione metabolism” (Ko00480), “Starch and sucrose metabolism” (Ko00500), “Glutathione metabolism” (Ko00480), “Steroid biosynthesis” (Ko00100), and “Flavonoid biosynthesis” (Ko00941) (Figure 4 and Table S6). In total, 55 and 18 DEGs were detected in the phenylpropanoid and flavonoid biosynthesis pathways, respectively. The aforementioned findings suggest that these genes may play a significant role in the determination of the diverse colours found in the leafstalks of the taro plant (Table S6). These results provide valuable information about anthocyanin accumulation in taro leafstalks. 

### 3.4. Identification of Potential Anthocyanin-Related Genes

**Figure 5:**
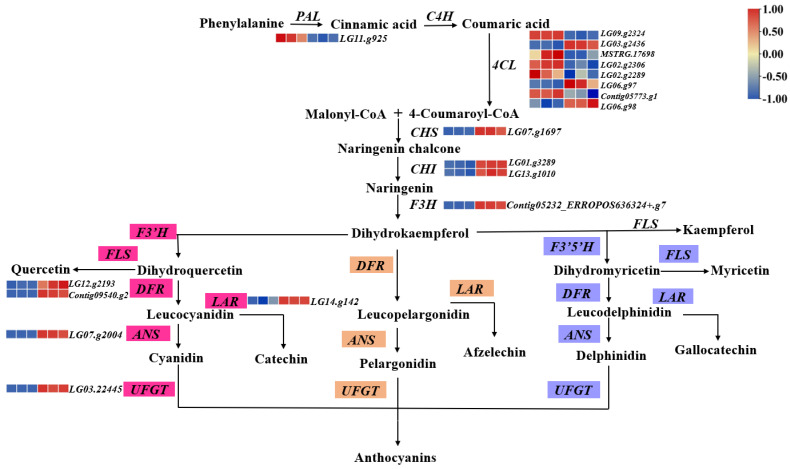
Pathway of anthocyanin biosynthesis in taro leafstalk. Pink, orange, and purple represent the biosynthetic pathways of different types of anthocyanins. The heat map next to the gene represents the expression level of the gene, with the blue colour representing low expression and the red colour representing high expression.

In this study, we investigated the genes responsible for encoding enzymes involved in pigment metabolism, with the aim of identifying DEGs associated with anthocyanin biosynthesis. The results revealed that 18 DEGs, including *PAL* (*LG11.g925*)*, 4CL* (*LG09.g2324, LG03.g2436, MSTRG.17698, LG02.g2306, LG02.g2289, LG06.g97, Contig05773.g1, LG06.g98*)*, CHS* (*LG07.g1697*)*, CHI* (*LG01.g3289, LG13.g1010*)*, F3H* (*Contig05232-ERROPOS636324+.g7*), *DFR* (*LG12.g2193, Contig09540.g2*), and *LAR* (*LG14.g142*), as well as *ANS* (*LG07.g2004*) and *UFGT* (*LG03.g2445*), showed elevated transcript abundance in purple taro leafstalk compared to in green taro leafstalk (Figure 5), whether they were in the beginning or end stages of the anthocyanin biosynthesis pathway.

### 3.5. Anthocyanin Biosynthesis-Related Transcription Factors

**Figure 6:**
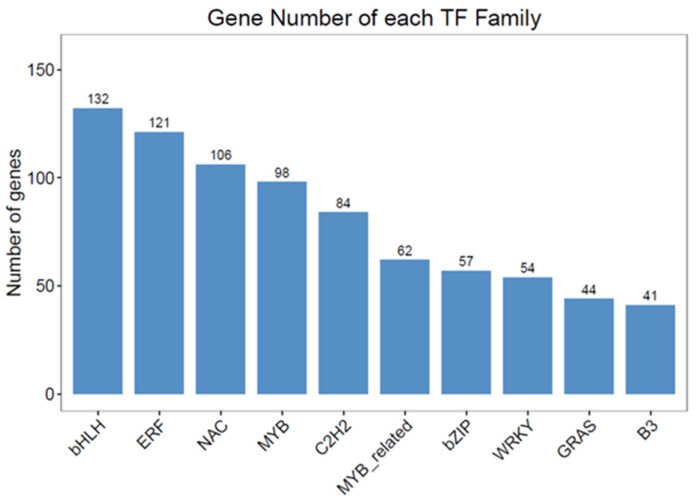
The number of differentially expressed top 10 transcription factors between green and purple taro leafstalks.

**Figure 7:**
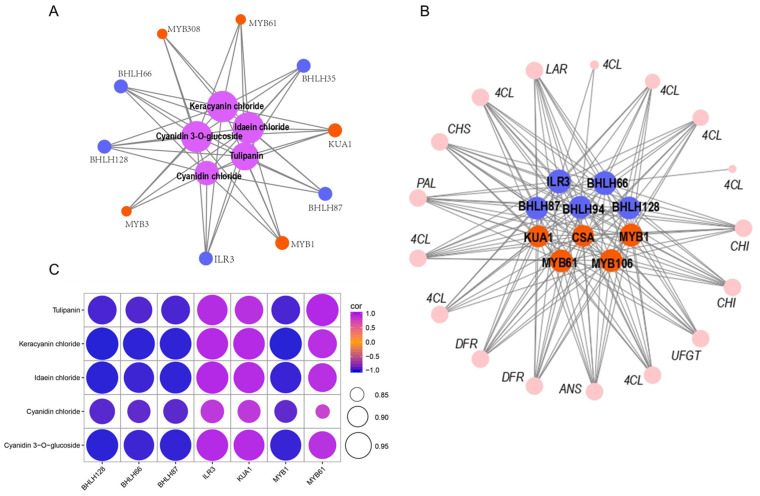
Association analysis of DEGs and anthocyanins. (**A**) Association analysis of anthocyanins with MYB and bHLH transcription factors. The purple-red circle in the figure represents 5 differentially expressed anthocyanins. Blue and orange circles represent the top 5 MYB and 5 bHLH transcription factors with the highest correlations, respectively. (**B**) Association analysis of anthocyanin structural genes with MYB and bHLH transcription factors. Pink circles represent structural genes, and blue and orange circles represent the top 5 MYB and bHLH transcription factors with the highest correlations, respectively. (**C**) Association analysis of anthocyanins and MYB and bHLH transcription factors with overlap in (**A**,**B**).

Transcriptional factors play a critical role in modulating anthocyanin accumulation because they regulate structural genes associated with anthocyanin biosynthesis. In this study, a total of 1192 transcription factors were identified based on transcriptome annotation. Classification results revealed that most of these TFs belonged to the ERF, bHLH, NAC, bZIP, C2H2, MYB, and WRKY families (Figure 6, Table S7). The top 5 MYB and 5 bHLH TFs (PCC > 0.9) implicated in the production of anthocyanin were determined by evaluating the PCC between the expression levels of these TFs and the total content of anthocyanin (Figure 7A). Additionally, using the same method, we also screened the top five MYB transcription factors closely linked to anthocyanin structure genes as well as bHLH transcription factors, respectively (Figure 7B). By comparing Figure 7A,B, seven overlapped transcription factors can be identified: three MYB (KUA1, MYB1, and MYB61) and four bHLHs (bHLH128, bHLH66, bHLH87, and ILR3). Their correlation with anthocyanins is shown in Figure 7C. Our findings suggest that these transcription factors may play a significant role in regulating anthocyanin biosynthesis. 

### 3.6. Verification of Gene Expression Profiles by qRT-PCR Analysis

**Figure 8:**
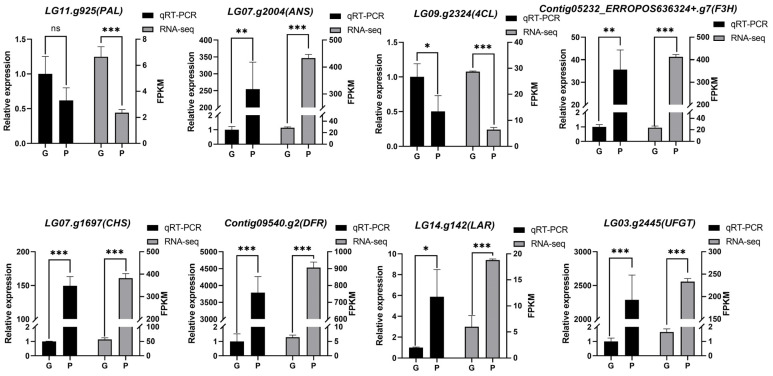
Verification of anthocyanin biosynthesis-related DEGs by qRT-PCR. Graphical representation of RNA-seq (right) and qRT-PCR (left) results. The *Y*-axis on the right side represents the FPKM (fragments per kilobase per million) obtained from transcriptome sequencing, and the *Y*-axis on the left side represents the relative expression of genes detected by qRT-PCR, while the *x*-axis shows the leafstalk samples: green taro leafstalk (G) and purple taro leafstalk (P). The results are presented as the average of three biological replicates ± SD. Asterisks denote *t*-test significance: ns, not significant, * *p* < 0.05; ** *p* < 0.01, *** *p* < 0.001.

To validate the transcriptome data, eight genes (*LG11.g925*, *LG07.g2004*, *LG09.g2324.*, *Contig05232-ERROPOS636324+.g7*, *LG07.g1697*, *Contig09540.g2*, *LG14.g142,* and *LG03.g2445*) involved in anthocyanin metabolism were chosen for verification using qRT-PCR. According to the qRT-PCR results, the transcript abundance patterns were nearly identical to those identified through the RNA-seq results (Figure 8).

## 4. Discussion

The taro leafstalk possesses significant nutritional value, rendering it a vital vegetable crop [1,41]. Purple vegetables have drawn increased attention because they contain more nutrients, especially anthocyanins.

Therefore, to understand the mechanism of anthocyanin biosynthesis in taro leafstalks, the present investigation used integrated metabolome and transcriptome analyses. There are several anthocyanin species found in colourful plants, of which six are well-established. Anthocyanins have been extensively investigated in terms of their molecular structure and chemical properties. According to a recent study, cyanidin is responsible for red-purple, pelargonidin for orange and red, and delphinidin for blue-red [42]. Various studies have shown that red vegetables contain cyanidin, a chromogenic substance. The vibrant red colour of cowpea pod is attributed to the presence of significant concentrations of cyanidin 3-O-glucosides and delphinidin 3-O-glucosides in its peel [43]. Red Mizuna contains 12 kinds of cyanidin glycosides compared with Green Mizuna [44]. One petunidin glycoside, one peonidin glucoside, and one cyanidin glycoside are more abundant in purple leaves of non-heading Chinese cabbage than in green leaves [3]. As a result of our study, we found a total of 75 differential metabolites from the metabolome (Figure 2A,B), as well as five anthocyanins that had differential expression (Figure 2C), including four cyanidin glycosides and one delphinidin glycoside. According to the results, cyanidin was mainly related to the taro leafstalk’s colour, which aligns with prior scholarly investigations which found that cyanidin is responsible for the red-purple colour. However, they are different in terms of cyanidin glycosides due to different red-purple crops and varieties. In addition, different shades of purple-red are also related to other anthocyanins, such as pelargonidin, delphinidin, and petunidin. In our study, we found that delphinidin was also differentially expressed between purple and green taro leafstalks (Figure 2C). Our results show that besides cyanidin, delphinidin is also responsible for taro colour.

Anthocyanin biosynthesis is regulated by numerous structural genes. Among the structural genes, purple plant tissues were the most highly expressed [43]. In the present study, we used transcriptomes to screen anthocyanin-related genes, and the KEGG enrichment results indicated that DEGs are mainly enriched in pathways related to anthocyanin biosynthesis (Figure 4). Moreover, the results of our transcriptome analysis showed that the expression of majority of structural genes involved in anthocyanin biosynthesis during the formation of purple stalks, in comparison to the green taro leafstalk, were increased (Figure 5). We drew a pathway of anthocyanin biosynthesis in taro leafstalks based on transcriptomes, and the result of qRT-PCR was consistent with this interpretation (Figure 8). This is also consistent with findings from a prior investigation of the vegetable [45]. The expression levels of *4CL* genes were demonstrated to be elevated in the purple taro leafstalk (Figure 5), which suggests that these genes are responsible for encoding upstream enzymes that regulate anthocyanin metabolism. In several plants, including *Vitis vinifera,* the activity of 4CL is significantly correlated with anthocyanin synthesis [46]. The condensation reaction of coumarin CoA and malonyl CoA catalysed by chalcone synthase (CHS) generates naringenin chalcone. In our study, the increased expression of the *CHS* gene in the purple taro leafstalk may have enhanced the synthesis of naringenin chalcone (Figure 5).

The purple taro leafstalk expressed higher levels of the *F3H* gene (Figure 5), proposing that *F3H* may potentially assume a significant function in facilitating the diversion of naringenin precursors toward the different anthocyanin pathways. The *F3H* functional genes are responsible for catalysing the synthesis of dihydroflavonol from substrates that are essential for the synthesis of both anthocyanins and proanthocyanidins, which are key components of all three branches of flavonoids [47]. Anthocyanin levels in strawberry fruit have been reported to decrease by approximately 70% upon the downregulation of the *F3H* gene through RNAi [48]. In the current investigation, it was observed that two *DFR* genes, namely, *LG12.g2193* and *Contig09540.g2*, together with one *ANS* gene, *LG07.g2004*, had greater transcript abundance in the purple taro leafstalk compared to the green taro leafstalk (Figure 5). In addition to the competitive interactions between enzymes at branch points, the substrate specificity of *DFR* plays a pivotal role in selecting the pathway that is pursued. *LhDFR* is only expressed in anthocyanin-stained tissues in *Asiatic hybrid lilies* [49]. Furthermore, ANS creates anthocyanins from leucoanthocyanidin by acting as an enzyme catalyst. According to one study, it was observed that the purple-red leaves of *Paeonia*×*suffruticosa* had a higher level of *ANS* expression compared to the yellow-green leaves [50]. Previous research has indicated that the overexpression of *SmANS* leads to an elevation in anthocyanin content in *Salvia miltiorrhiza*; conversely, the decreased transcript abundance of *ANS* in white flowers of *S. miltiorrhiza* has been associated with their white pigmentation [51,52]. The upregulation of *ANS* could stimulate anthocyanin accumulation in purple taro leafstalks, which is consistent with the above research. As DFR is the precursor to ANS, they can work together to synthesise cyanidin, and it has been hypothesised that higher levels of ANS and DFR activity may result in more cyanidin production for anthocyanin synthesis [53,54]. It is postulated that the heightened conversion of dihydroflavonol to cyanidin is facilitated by the precise amplification of the *DFR* and *ANS* genes, which is consistent with the metabolomic analysis in that purple stalks contain a large amount of cyanidin.

The MYB and bHLH transcription factors are important for anthocyanin production in plants. The anthocyanin biosynthesis pathway and skin colour are greatly controlled by the R2R3 MYB transcription factor PavMYB10.1 in sweet cherry fruit [55]. Under light stress, the accumulation of anthocyanin depends on MYB75 phosphorylation by MPK4 in Arabidopsis [56]. The transcript levels of R2R3-MYB *MdMYB10* alleles in apple (*Malus × domestica* Borkh.) have been found to contribute to a higher accumulation of anthocyanin, and the accumulation was greater in red cultivars than in green-fruited cultivars [57,58,59]. In Arabidopsis, overexpression of *DcMYB6* leads to a significant increment in anthocyanin content in both vegetative and reproductive tissues, as well as to the upregulation of expression of all seven anthocyanin-regulated structural genes [60]. In apricots, PaMYB10 functions as an important transcription factor and plays a pivotal role in anthocyanin synthesis [61]. Furthermore, in plants, the production of flavonoids is greatly influenced by bHLH transcription factors. The promoters of *PsANS* and *PsDFR* are directly activated by PsbHLH in peonies, resulting in controlled biosynthesis of anthocyanin [62]. *F3H* and *DFR* transcriptional levels were significantly upregulated in *Centaurea cyanus* by CcbHLH6-1 [63]. In our study, we identified three MYB transcription factors and four bHLH transcription factors based on integration of transcription factors and structure gene association analyses as well as transcription factor and anthocyanin association analyses (Figure 7). These seven transcription factors showed significant variations in expression in two taro leafstalk varieties with different flavonoid contents and components, demonstrating that the transcription factors MYB and bHLH might play regulatory roles in flavonoid synthesis in taro leafstalks. 

## 5. Conclusions

To the best of our knowledge, this work is the first to thoroughly examine the transcriptomes and metabolomes of both green and purple taro leafstalks to determine the expression of genes and the composition of various metabolites. The colour variations are attributed to modulations of anthocyanin metabolites, in particular cyanidin production in purple plants. Additionally, 3 MYB TF and 4 bHLH TF were identified, which are closely related to anthocyanin biosynthesis in purple taro leafstalks. In this study, we gained a deeper understanding of flavonoid-related metabolites in purple taro leafstalks and established significant reference values for the purpose of improving the colouration of purple taro leafstalks. The colour change in taro may be the result of natural selection or human selection. Further studies are needed in order to find out why taro leafstalks acquire a purple colour. Genes are carriers of genetic information. By comparing plant genes and genomes, the genetic mechanisms and variation patterns of plant evolution can be revealed.

## Data Availability

The data presented in this study are available upon request from the corresponding author.

## Supplementary Materials

The following supporting information can be downloaded at: https://www.mdpi.com/article/10.3390/genes15010138/s1. Figure S1: PCA analysis, heat map cluster analysis, and sample correlation analysis of all flavonoid metabolites; Figure S2: Sample correlation analysis and PCA analysis of purple and green taro leafstalk; Table S1: Primers used in this study; Table S2: The DAMs detected in this study; Table S3: Sequencing data statistics; Table S4: DEGs in this study; Table S5: The GO terms enriched by the DEGs; Table S6: The KEGG enriched pathway by the DEGs; Table S7: TF detected in this study.
