# Supplementary material for: Comparative Metabolome and Transcriptome Analyses Reveal the Regulatory Mechanism of Purple Leafstalk Production in Taro (Colocasia esculenta L. Schott)
Source: Genes (Basel). 2024 Jan 22;15(1):138. doi: 10.3390/genes15010138 (PMC10815928; doi:10.3390/genes15010138)
Supplement: Supplementary file 1 [file genes-15-00138-s001.zip › supplementary Figure.pdf]

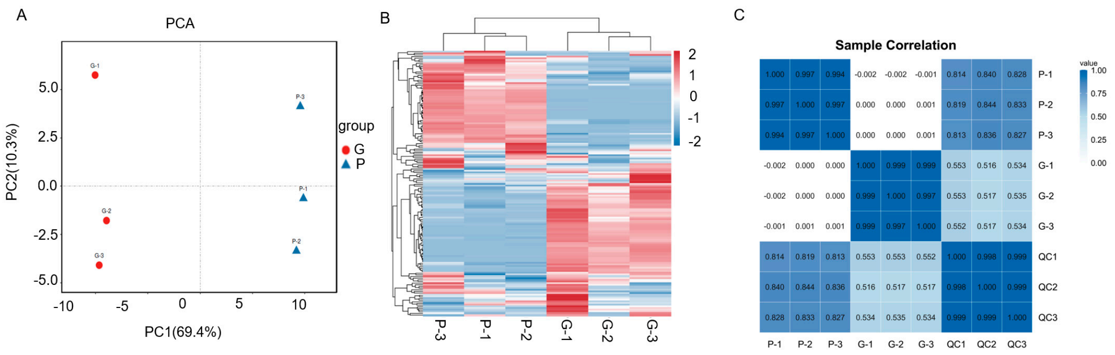

Figure S1. PCA analysis, heat map cluster analysis, and sample correlation analysis of all flavonoid metabolites.

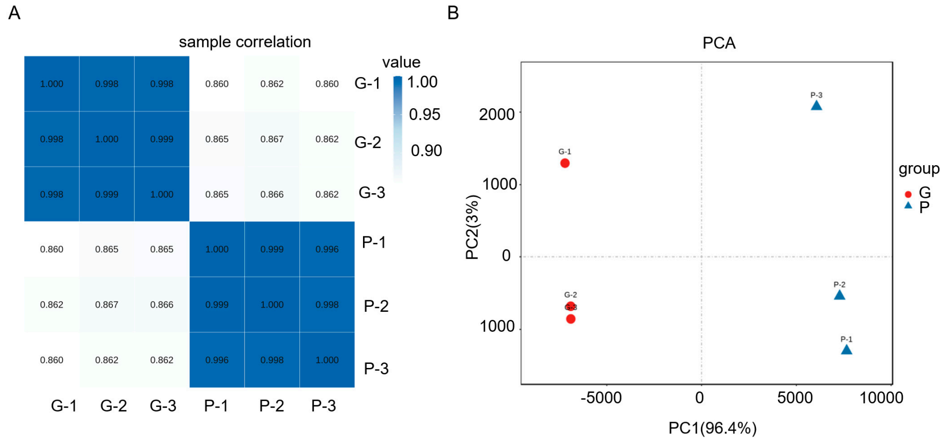

Figure S2. Sample correlation analysis and PCA analysis of purple and green taro leafstalk.
